# Latitudinal variation in ecological opportunity and intraspecific competition indicates differences in niche variability and diet specialization of Arctic marine predators

**DOI:** 10.1002/ece3.1980

**Published:** 2016-02-14

**Authors:** David J. Yurkowski, Steve Ferguson, Emily S. Choy, Lisa L. Loseto, Tanya M. Brown, Derek C. G. Muir, Christina A. D. Semeniuk, Aaron T. Fisk

**Affiliations:** ^1^ Great Lakes Institute for Environmental ResearchUniversity of WindsorWindsorONN9B 3P4Canada; ^2^ Freshwater InstituteFisheries and Oceans CanadaWinnipegMBR3T 2N6Canada; ^3^ Department of Biological SciencesUniversity of ManitobaWinningMBR3T 2N6Canada; ^4^ Department of GeographyMemorial University of NewfoundlandSt. John'sNFA1B 3X9Canada; ^5^ Environment CanadaAquatic Ecosystem Protection Research DivisionBurlingtonONL7R 4A6Canada

**Keywords:** Beluga whale, generalist, marine mammals, ringed seal, stable isotopes, trophic ecology

## Abstract

Individual specialization (IS), where individuals within populations irrespective of age, sex, and body size are either specialized or generalized in terms of resource use, has implications on ecological niches and food web structure. Niche size and degree of IS of near‐top trophic‐level marine predators have been little studied in polar regions or with latitude. We quantified the large‐scale latitudinal variation of population‐ and individual‐level niche size and IS in ringed seals (*Pusa hispida*) and beluga whales (*Delphinapterus leucas*) using stable carbon and nitrogen isotope analysis on 379 paired ringed seal liver and muscle samples and 124 paired beluga skin and muscle samples from eight locations ranging from the low to high Arctic. We characterized both within‐ and between‐individual variation in predator niche size at each location as well as accounting for spatial differences in the isotopic ranges of potential prey. Total isotopic niche width (TINW) for populations of ringed seals and beluga decreased with increasing latitude. Higher TINW values were associated with greater ecological opportunity (i.e., prey diversity) in the prey fish community which mainly consists of Capelin (*Mallotus villosus*) and Sand lance (*Ammodytes* sp.) at lower latitudes and Arctic cod (*Boreogadus saida*) at high latitudes. In beluga, their dietary consistency between tissues also known as the within‐individual component (WIC) increased in a near 1:1 ratio with TINW (slope = 0.84), suggesting dietary generalization, whereas the slope (0.18) of WIC relative to TINW in ringed seals indicated a high degree of individual specialization in ringed seal populations with higher TINWs. Our findings highlight the differences in TINW and level of IS for ringed seals and beluga relative to latitude as a likely response to large‐scale spatial variation in ecological opportunity, suggesting species‐specific variation in dietary plasticity to spatial differences in prey resources and environmental conditions in a rapidly changing ecosystem.

## Introduction

Food web models are typically studied at the species level where trait variation among individuals is often not incorporated (Miller and Rudolf 2011). However, it is also widely accepted in the ecological literature that substantial dietary variation exists among individuals of a given species or population (Rudolf and Lafferty 2011). Species that consume a wide range of resources are considered generalists, a relative term that compares species, but may actually be composed of individual dietary specialists with each consuming a small subset of resources that differs across individuals (Bolnick et al. 2003). As such, these individual specialists may have different ecological roles in terms of their habitat use and feeding relationships within an ecosystem. Thus, individual specialists may be more susceptible to ecosystem perturbations such as changing prey diversity and abundance, than generalist ones (Miller and Rudolf 2011).

Based on the niche variation hypothesis (Van Valen 1965), Bolnick et al. (2003) introduced the concept of individual specialization (IS) which occurs when individuals irrespective of age, sex, and body size have a significantly narrower niche using a small subset of resources than those of the population's total niche width (TNW). Individual specialization in resource use is prevalent among animal taxa (Araújo et al. 2011) and has several important implications for understanding the complexity of food webs by contributing another mechanism to ecosystem trophodynamics (Quevedo et al. 2009). The causes of IS include interspecific and intraspecific competition for resources, ecological opportunity (*i.e.,* prey diversity), and predation where all factors are, at some level, influenced by prey species richness and abundance (Araújo et al. 2011). For example, based on an optimal foraging theory, a decrease in the abundance of preferred prey can increase intraspecific competition causing the population to broaden their diet and increase their ecological niche size potentially leading to a higher degree of IS among individuals (Kernaléguen et al. 2015). Similarly, increased prey diversity can increase the ecological niche size for consumers, possibly leading to a higher degree of IS among individuals (Darimont et al. 2009).

Individual specialization has mainly been documented in animal species inhabiting tropical and temperate ecosystems (Araújo et al. 2011) with only a handful of studies investigating it in the Arctic (Woo et al. 2008; Thiemann et al. 2011; Dalerum et al. 2012; Tarroux et al. 2012; Provencher et al. 2013) – an ecosystem with the lower levels of species richness than temperate and tropical systems (MacArthur 1955). The low Arctic marine environment has more biodiversity than the high Arctic (Bluhm et al. 2011) with at least double the amount of species richness from 60° to 75°N (Cheung et al. 2009) and in Hudson Bay relative to the rest of the Canadian Arctic (Archambault et al. 2010), allowing higher trophic‐level arctic species to have more opportunity to broaden their diet and expand their ecological niche at the lower latitudes. As a result of climate change, many non‐native, forage fish species in the Arctic, such as Capelin (*Mallotus villosus*), Sand lance (*Ammodytes* sp.), and Walleye Pollock (*Theragra chalcogramma*; Wassmann et al. 2011; Provencher et al. 2012), as well as pelagic plankton are now prevalent which may further increase differences in IS and ecological niche sizes between low and high Arctic predator populations. This northward expansion of subarctic species is predicted to continue, as up to 44 subarctic fish species are predicted to traverse the Northwest and Northeast Passages via the Atlantic and Pacific Oceans by 2100 (Wisz et al. 2015).

**Figure 1:**
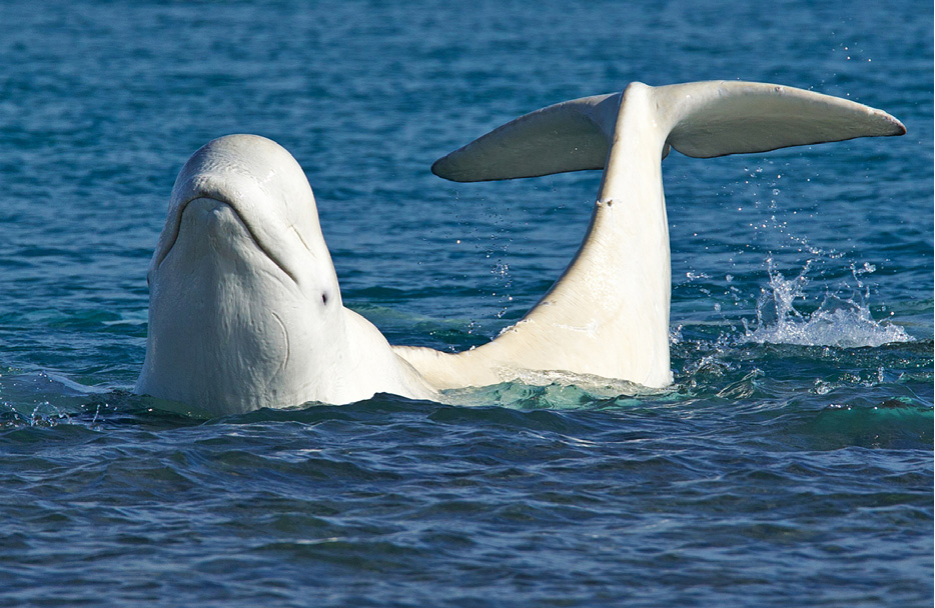
Beluga whale in Cunningham Inlet, Nunavut, Canada. Photograph courtesy of Gretchen Freund.

Ringed seals (*Pusa hispida*) and beluga whales (*Delphinapterus leucas*; Fig. 1) are higher trophic‐level predators (Hobson and Welch 1992) that inhabit a wide diversity of habitats in the Arctic, from shallow coastal zones and estuaries to deep ocean basins (Laidre et al. 2008). Ringed seals and beluga have a circumpolar distribution and are thought to be the most abundant pinniped and cetacean species in the Arctic, albeit with abundances varying spatially and an unknown total species abundance (Laidre et al. 2015). Ringed seals consume a wide variety of prey from zooplankton to fish (Thiemann et al. 2007; Chambellant et al. 2013), which varies with age, space (Yurkowski et al. in press), and season (Young and Ferguson 2013). Beluga whales mainly consume pelagic forage fish, such as Arctic cod (*Boreogadus saida*; Loseto et al. 2009), but have been documented to consume squid (Quakenbush et al. in press) and benthic fishes and crustaceans (Marcoux et al. 2012). Given the high abundance, wide distribution, and diverse diets of ringed seals and beluga, both species are excellent models to investigate the ecological niche width and degree of IS relative to ecological opportunity and intraspecific competition in arctic species and how this varies with latitude.

In this study, we used a unique dataset consisting of stable carbon (*δ* ^13^C) and nitrogen (*δ* ^15^N) isotope ratios of ringed seal liver and muscle and beluga whale skin and muscle to quantify individual‐ and population‐level niche variation in terms of WIC, BIC, TNW and the degree of IS relative to latitude, longitude, and ringed seal density across the Arctic. Stable isotope analysis provides data on what an animal consumes and the habitat within which it resides and is commonly used to determine an animal's ecological niche (Bearhop et al. 2004). In addition, tissues of a consumer incorporate the isotopic composition of their prey at different rates depending on tissue‐specific metabolic turnover rates; thus, stable isotope analysis of different body tissues provides time‐integrated dietary information (Thomas and Crowther 2015) and has become a robust tool when investigating intra‐individual and interindividual niche variation (Layman et al. 2012). The metabolic rate of larger body‐sized mammalian skin and liver is higher than muscle, resulting in shorter stable isotope half‐lives in skin and liver than muscle (Vander Zanden et al. 2015). Thus, both liver and skin can be used as short‐term indicators of diet, whereas muscle is a longer‐term indicator, providing the necessary temporal scope to examine the individual specialization using multiple tissues (Araújo et al. 2007). The total variance of *δ* ^13^C and *δ* ^15^N between individuals in a population represents BIC, and the variance of *δ* ^13^C and *δ* ^15^N values between tissues within an individual illustrates dietary variation or consistency for that particular individual over time (*i.e.,* WIC; Newsome et al. 2009). The sum of both components represents TINW (Newsome et al. 2009). We hypothesized that due to higher ecological opportunity in the low Arctic relative to the high Arctic, the total niche width and degree of IS of ringed seals and beluga whales will be higher at lower latitudes, aligning with optimal foraging theory (MacArthur and Pianka 1966). In addition, we hypothesized that in locations with the highest density estimates for both species, total niche width and the degree of IS will be highest due to intraspecific competition.

## Materials and Methods

### Sample collection and preparation

**Figure 2:**
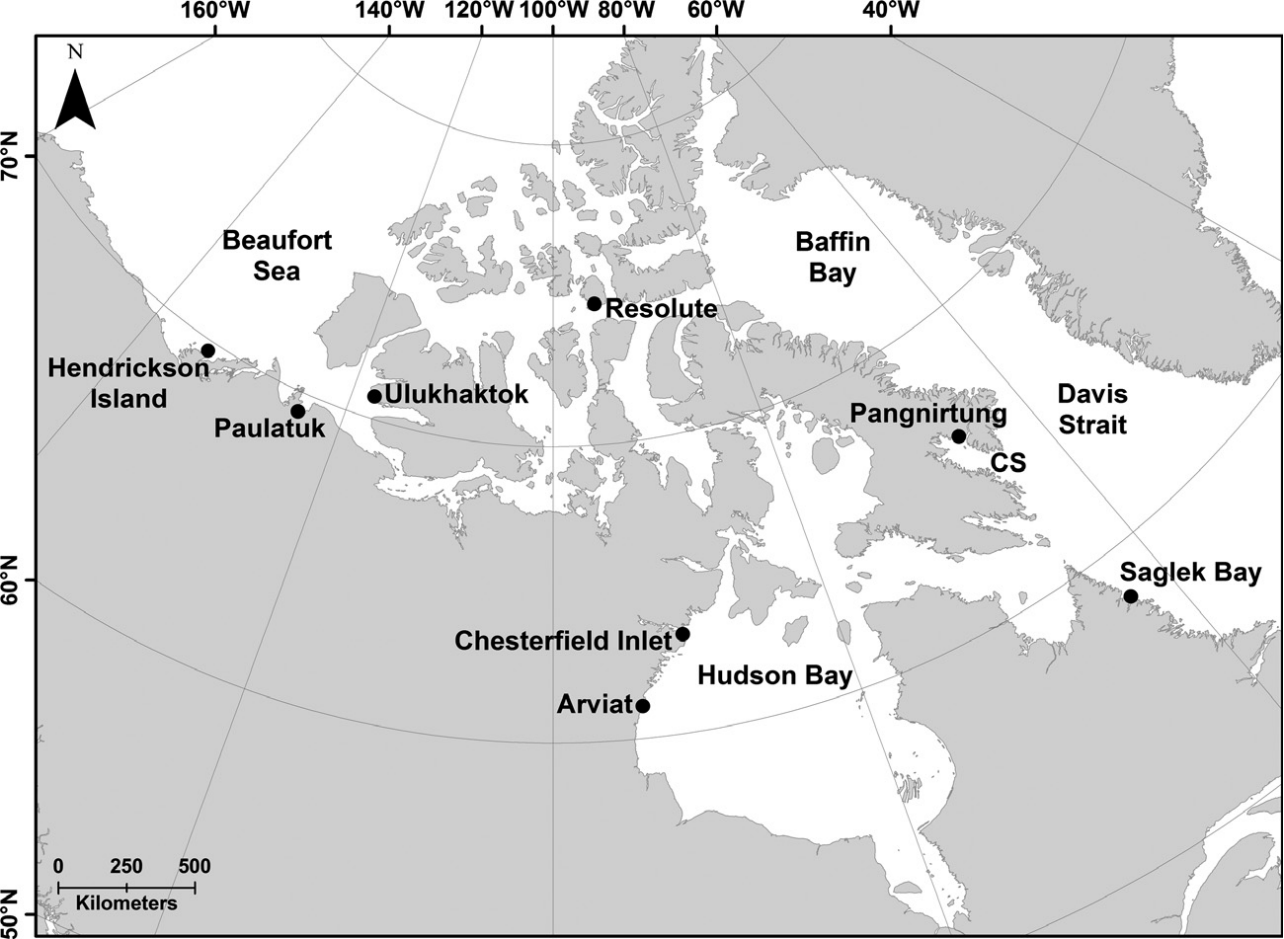
Map of locations where ringed seal liver and muscle samples and beluga whale skin and muscle samples were collected for stable isotope analysis. See Table 1 for sample sizes. CS: Cumberland Sound

Paired ringed seal liver and muscle and beluga whale skin and muscle were collected opportunistically by Inuit hunters across the Canadian Arctic as a part of their summer (June to September) subsistence harvests from 1986 to 2012 (Fig. 2). These opportunistic collections are in context of the community‐based monitoring program coordinated by the Department of Fisheries and Oceans Canada in Winnipeg, Manitoba, Canada, and Environment Canada in Burlington, Ontario, Canada. A total of 379 ringed seals with paired liver and muscle samples (see Table 1 for samples sizes by location) were analyzed for δ^13^C and δ^15^N. With the spatial scope of the study, locations across the Arctic for both species represent distinct foraging groups, as the distribution of beluga populations generally remains nearby sampling locations throughout the summer period at all locations (see Hauser et al. 2014 for Beaufort Sea beluga; Koski and Davis 1980 for Resolute beluga; DFO 2013 for Cumberland Sound beluga; and Richard 2005 for Western Hudson Bay beluga). Similarly, ringed seal distribution and movements during the summer are generally nearby and within sampling locations (see Luque et al. 2014 for Hudson Bay ringed seals; Brown et al. (2014) for Saglek Bay ringed seals; Harwood et al. 2015 for Ulukhaktok ringed seals; D. J. Yurkowski unpubl. data for other locations).

**Table 1:** Sample sizes of paired ringed seal liver and muscle, and beluga whale skin and muscle by age class, sex, and location used for stable isotope analysis

Location	Year	Adult		Subadult	
		Male	Female	Male	Female
Ringed seal					
Resolute	2004–2012	24	10	8	4
Ulukhaktok	1995–2010	97	44	2	10
Pangnirtung	1990–2009	17	18	23	19
Chesterfield Inlet	1999–2000	12	16	4	2
Saglek Bay	2008–2011	28	31	5	5
Beluga					
Resolute	1999–2009	8	3	–	–
HI/Paulatuk	2011–2012	32	–	–	–
Pangnirtung	1986–2006	13	7	7	4
Arviat	2003–2008	20	11	8	4

HI, Hendrickson Island.

Individual ringed seals were grouped into two age classes based on age of sexual maturity: (1) adults ≥6 years of age and (2) subadults 1–5 years of age (McLaren 1958) via counting annual growth layer groups (GLG) in the cementum of decalcified, stained, and longitudinal thin sections of the lower right canine for individuals collected in Pangnirtung, Resolute, Saglek Bay, and Chesterfield Inlet. Ringed seals collected in Ulukhaktok were aged by counting GLG in the dentine layer of canine teeth from the lower right canine, which can underestimate ages of seals over 10 years of age (Stewart et al. 1996), but will have no effect on our results due to the age class groupings. The ages of beluga were estimated by counting GLGs in the dentine of teeth extracted from the mandible, and individuals were divided into two age groups based on age of sexual maturity (subadults ≤11 years of age and adults >11 years of age), similar to those of Marcoux et al. (2012). Standard lengths (cm) were measured as the straight‐line distance from the tip of the nose to the end of the tail in ringed seals and from the tip of the head to the tail fork in beluga (American Society of Mammalogists 1961).

We include the ranges of mean *δ* ^13^C and *δ* ^15^N values of potential prey items for beluga (Loseto et al. 2009) and ringed seals (Yurkowski et al. in press) from the benthic and pelagic environments, including zooplankton, shrimp, and fish to account for spatial variation in the absolute stable isotope values and ranges among prey sources (Table 2), which, when unaccounted for, can confound the interpretations of WIC (Matthews and Mazumber 2004). The stable isotope values from potential prey items included *Calanus* sp., *Themisto libellula*,* euphausiids*, benthic shrimp, Arctic cod, Capelin, Sand lance, and Sculpin (see Table 2 for *δ* ^13^C and *δ* ^15^N ranges of prey sources). Prey items were collected during the Arctic summer months (June to September) via nets and trawls at each location from 2003 to 2012.

**Table 2:** Variance component analysis from linear mixed‐model analysis for ringed seal and beluga *δ* ^13^C and *δ* ^15^N values at each location. Total niche width is the sum of the intercept and residual variances for *δ* ^13^C and *δ* ^15^N at each location. Total intercept variance (BIC) and total residual variance (WIC) are calculated by combining the intercept variances for *δ* ^13^C and *δ* ^15^N and then divided by total niche width (TINW) at each location. Greater total intercept variances than total residual variances are highlighted in bold indicating a group of individual specialists. Proportion of WIC and BIC that explained TINW is in parentheses

Location	*δ* ^13^C (‰)			*δ* ^15^N (‰)			Total	Total	TINW
	Intercept Variance	Residual Variance	Conditional *r* ^2^	Intercept Variance	Residual Variance	Conditional *r* ^2^	Intercept Variance (%)	Residual Variance (%)	
Ringed seal									
Resolute	0.10	0.11	0.74	0.16	0.33	0.46	0.26 (37)	0.44 (63)	0.70
Ulukhaktok	0.06	0.16	0.33	0.18	0.17	0.65	0.24 (42)	0.33 (58)	0.57
Pangnirtung	**0.23**	0.09	0.81	0.39	0.39	0.58	**0.62 (59)**	0.48 (41)	1.10
Chesterfield Inlet	**0.30**	0.10	0.85	**0.97**	0.24	0.85	**1.27 (79)**	0.34 (21)	1.61
Saglek Bay	0.10	0.43	0.33	0.44	0.43	0.67	0.54 (39)	0.86 (61)	1.40
Beluga									
Resolute	0.04	0.04	0.67	0.00	0.15	0.13	0.04 (17)	0.19 (83)	0.23
HI/Paulatuk	0.08	0.13	0.83	0.05	0.14	0.83	0.13 (33)	0.27 (67)	0.40
Pangnirtung	0.05	0.02	0.93	0.01	0.40	0.82	0.06 (13)	0.42 (87)	0.48
Arviat	0.15	0.46	0.61	0.00	1.73	0.40	0.15 (6)	2.19 (94)	2.34

HI, Hendrickson Island.

### Stable isotope analysis

Frozen tissue samples were freeze‐dried for 48 h and then crushed into a fine powder using a mortar and pestle. Due to the effects of lipids on *δ* ^13^C values in Arctic marine mammal tissues (Yurkowski et al. 2015), lipids were extracted using a 2:1 chloroform:methanol similar to the Bligh and Dyer (1959) method, and subsequently, 400–600 *μ*g of tissue was weighed into tin capsules for analysis. Prey samples (Table 2) have also been lipid extracted to reduce the interindividual and species differences in lipid content to provide comparable *δ* ^13^C values between species and standardize the range of *δ* ^13^C values between prey items among locations. The *δ* ^15^N and *δ* ^13^C values from ringed seal and beluga tissues were measured by a Thermo Finnigan Delta^Plus^ mass‐spectrometer (Thermo Finnigan, San Jose, CA, USA) coupled with an elemental analyzer (Costech, Valencia, CA, USA) at the Chemical Tracers Laboratory, Great Lakes Institute for Environmental Research, University of Windsor. A triplicate was run for every 10th sample, and a measurement precision for *δ* ^13^C and *δ* ^15^N was 0.1‰ and 0.1‰, respectively. The analytical precision derived from the standard deviation of replicate analyses of a NIST standard (NIST 8414, *n* = 194) and an internal laboratory standard (tilapia muscle, *n* = 194) was both 0.1‰ and <0.1‰ for *δ* ^15^N and *δ* ^13^C, respectively. Beluga muscle samples from Arviat (*n* = 43) were lipid extracted, weighed at 1 mg into tin capsules, and then analyzed for *δ* ^13^C and *δ* ^15^N at the University of Winnipeg on a GV‐Instruments IsoPrime mass spectrometer (Wythenshave, Manchester, UK) attached to an elemental analyzer (EuroVector, Milan, Italy) where a duplicate was run for every 10th sample for a measurement precision of 0.2‰ for both *δ* ^13^C and *δ* ^15^N. Beluga skin and muscle samples from Hendrickson Island and Paulatuk (*i.e.,* near the Beaufort Sea) were lipid extracted, 1 mg of tissue weighed into tin capsules, and then, *δ* ^13^C and *δ* ^15^N were analyzed at the University of Waterloo on a Thermo Finnigan Delta^Plus^ XL mass spectrometer (Thermo Finnigan, Bremen, Germany) equipped with an elemental analyzer (Carlo Erba, Milan, Italy) where a duplicate was run every 10th sample for a measurement precision of 0.1‰ for both *δ* ^13^C and *δ* ^15^N. Analytical precision of international reference material (IAEA‐N1+ N2, IAEA‐CH3+ CH6) was <0.2‰ for *δ* ^13^C and <0.3‰ for *δ* ^15^N. Stable isotope ratios are expressed in parts per thousand (‰) in delta (*δ*) notation using the following equation: *δX* = [(*R*_sample_/*R*_standard_) − 1] × 1000, where *X* is ^13^C or ^15^N and R equals ^13^C/^12^C or ^15^N/^14^N. The standard material for ^13^C and ^15^N is Pee Dee Belemnite and atmospheric nitrogen, respectively.

### Data analysis

To eliminate the influence of tissue‐specific differences in stable isotope values relative to diet and allow the direct comparisons between liver and muscle, we corrected *δ* ^13^C and *δ* ^15^N values in ringed seal liver and muscle using known diet–tissue discrimination factors (DTDFs) in phocids (1.3‰ and 0.6‰ for *δ* ^13^C in liver and muscle, respectively, and 3.1‰ and 2.4‰ for *δ* ^15^N in liver and muscle, respectively; Hobson et al. 1996). The DTDFs used for beluga were reported values in other cetacean species where 1.3‰ was used for *δ* ^13^C and 1.2‰ for *δ* ^15^N in muscle (Caut et al. 2011) and 2.4‰ for *δ* ^13^C and 3.2‰ for *δ* ^15^N in skin (Browning et al. 2014).

We used linear mixed models at each location to assess the effects of age class, sex, standard body length, tissue type, and year collected (to account for interannual variation in stable isotope values) on ringed seal and beluga *δ* ^13^C and *δ* ^15^N values (run separately by species and element) with sample ID as a random effect. Categorical fixed factors included age class (adult and subadult), sex (female and male), and tissue (liver or skin, and muscle), whereas standard body length and year collected were continuous fixed factors. Tissue type represented the categorical time period of isotopic turnover for liver and skin (*i.e.,* short‐term diet indicator) and muscle (*i.e.,* long‐term diet indicator) to allow the repeated measures from each individual. For each population and element, we used mixed‐model variance component analysis in the random effect (*i.e.,* sample ID) term to estimate the total observed variability (*i.e.,* total isotopic niche width – TINW) for the population by summing the intercept variability (between‐individual component – BIC) representing dietary variability between individuals and residual variability (*i.e.,* within‐individual component – WIC; Roughgarden 1972; Newsome et al. 2009), representing dietary consistency of an individual over time. Variance components for *δ* ^13^C and *δ* ^15^N of each population were then summed following Newsome et al. (2009). A higher BIC than WIC would be more indicative of a specialist population, whereas a higher WIC would signify a generalist population. The degree of IS is represented by the WIC/TNW ratio where values closer to 0 represent an increased degree of individual specialization (Newsome et al. 2009), and values ≥0.5 represent generalization (Hückstädt et al. 2012). Stable isotope values from ringed seals, beluga, and their prey do not need to be corrected for baseline nor temperature changes with latitude as we are not comparing absolute stable isotope values between locations, but rather variation within and between individuals at each location for each species. We then used linear regression to determine the relationships between WIC, BIC, TINW, and WIC/TINW with latitude and longitude. Statistical analyses were performed in R v. 3.1.1 (R Development Core Team 2015) using the nlme package v. 3.1‐118 (Pinheiro et al. 2015) with an *α* of 0.05.

## Results

Results from linear mixed‐model analyses revealed a significant effect on DTDF‐corrected *δ* ^13^C and *δ* ^15^N values related to tissue type and standard length across all locations for ringed seals (Appendix S1). A significant relationship between *δ* ^15^N and age class occurred in Pangnirtung, Resolute, Saglek Bay, and Ulukhaktok, whereas a significant relationship between *δ* ^13^C and age class only occurred in Pangnirtung (Appendix S1). Year of collection had a significant effect on *δ* ^15^N in Saglek Bay and Ulukhaktok, whereas sex only had a significant effect on *δ* ^15^N in Ulukhaktok. In beluga whales, tissue type had the most significant effect on both DTDF‐corrected *δ* ^13^C and *δ* ^15^N followed by year and standard length for *δ* ^15^N in Pangnirtung and sex for *δ* ^15^N in Arviat (Appendix S2).

Results from mixed‐model variance component analysis revealed that total intercept variance (*i.e.,* BIC) accounted for 59% and 79% of TINW in Pangnirtung and Chesterfield Inlet, respectively, indicating that ringed seals inhabiting these areas are composed of individual specialists (Table 2). In contrast, total residual variance accounted for most of the variations in stable isotope values for ringed seals in Resolute, Ulukhaktok, and Saglek Bay, and beluga whales from all locations, ranging from 58% in Ulukhaktok ringed seals to 88% in Pangnirtung beluga, suggesting dietary generalization for each of these populations (Table 2). The *δ* ^13^C and *δ* ^15^N ranges of prey items across locations were similar (Table 3). This suggests that isotopic variation between pelagic and benthic energy pathways and isotopic variation between zooplankton and fish prey items across locations were similar allowing comparison in WIC, BIC, and TINW metrics between locations.

**Table 3:** Mean stable isotope value ranges between benthic and pelagic (*δ* ^13^C) prey and invertebrate to fish (*δ* ^15^N) prey for ringed seals and beluga whales at each location

Location	Range of mean *δ* ^13^C values of prey (‰)	Range of mean *δ* ^15^N values of prey (‰)	Source
Resolute	−21.4 to −17.0 (4.4)	8.7 to 14.6 (5.9)	1
Amundsen Gulf	−26.1 to −21.5 (4.6)	9.4 to 14.7 (5.3)	2
Pangnirtung	−20.8 to −16.8 (4.0)	9.0 to 15.6 (6.4)	3 and 4
Hudson Bay	−22.7 to −18.0 (4.7)	9.7 to 14.7 (5.0)	5
Saglek Bay	−20.4 to −17.0 (3.4)	8.5 to 14.4 (5.9)	1 and this study

Sources include the following: (1) Yurkowski et al. (in press), (2) Loseto et al. (2008), (3) Marcoux et al. (2012), (4) McMeans et al. (2013), and (5) Chambellant et al. (2013). The mean *δ* ^13^C and *δ* ^15^N values of *Calanus* sp. (*n* = 43) collected from Saglek Bay were −20.4 ± 0.6‰ (mean ± SD) and 9.8 ± 0.4‰, respectively.

**Figure 3:**
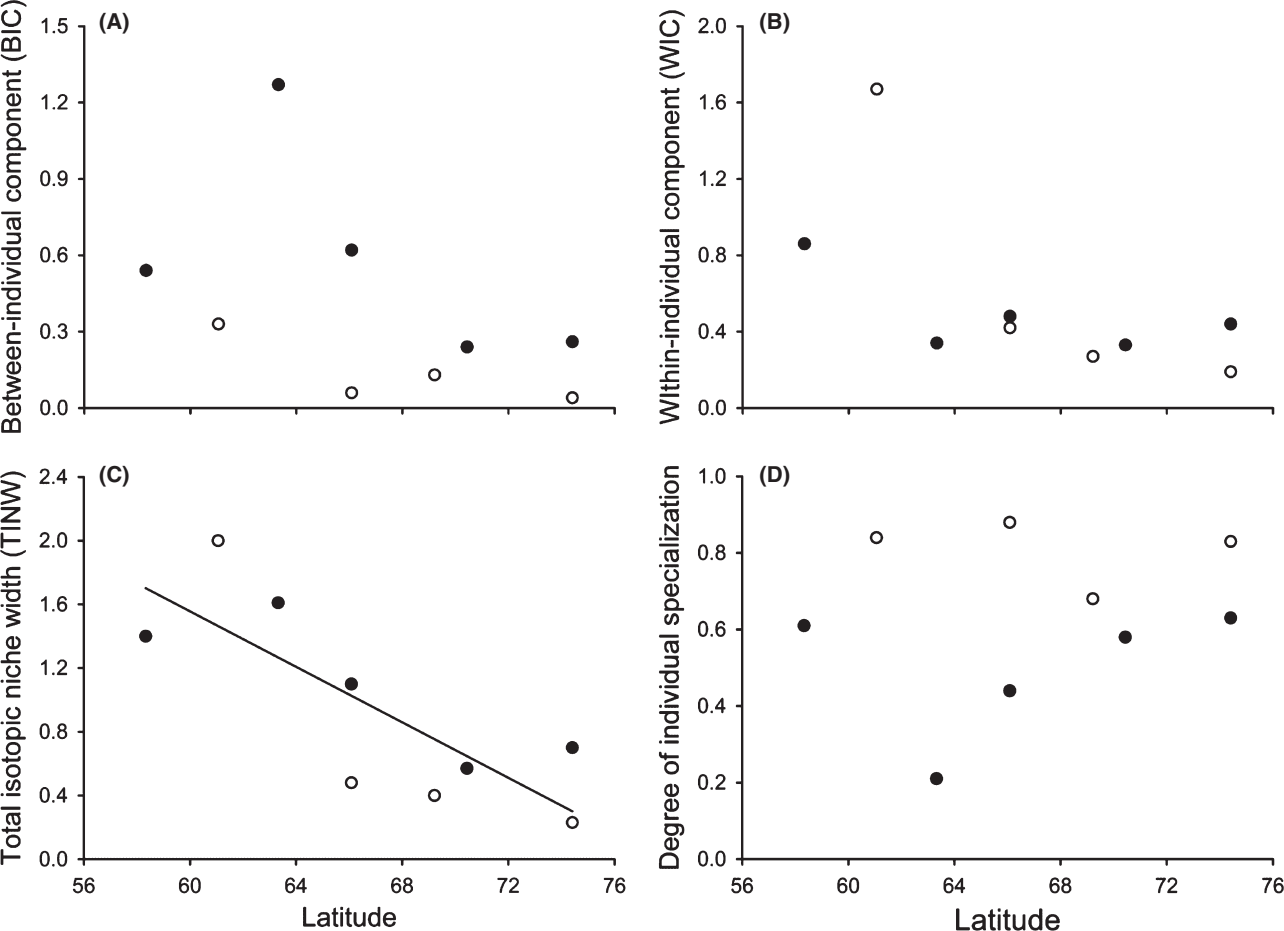
Linear regressions of (A) between‐individual component (BIC), (B) within‐individual component (WIC), (C) total isotopic niche width (TINW), and (D) degree of individual specialization (WIC/TINW) for combined ringed seals (closed circles) and beluga whales (open circles) relative to latitude. A significant relationship only occurred between TINW and latitude (C, slope = −0.09, *r* ^2^ = 0.64, *F*_1,8_ = 12.24, *P* < 0.01) when both species were analyzed together. No significant relationships between each niche metric and latitude occurred when species were analyzed separately.

**Figure 4:**
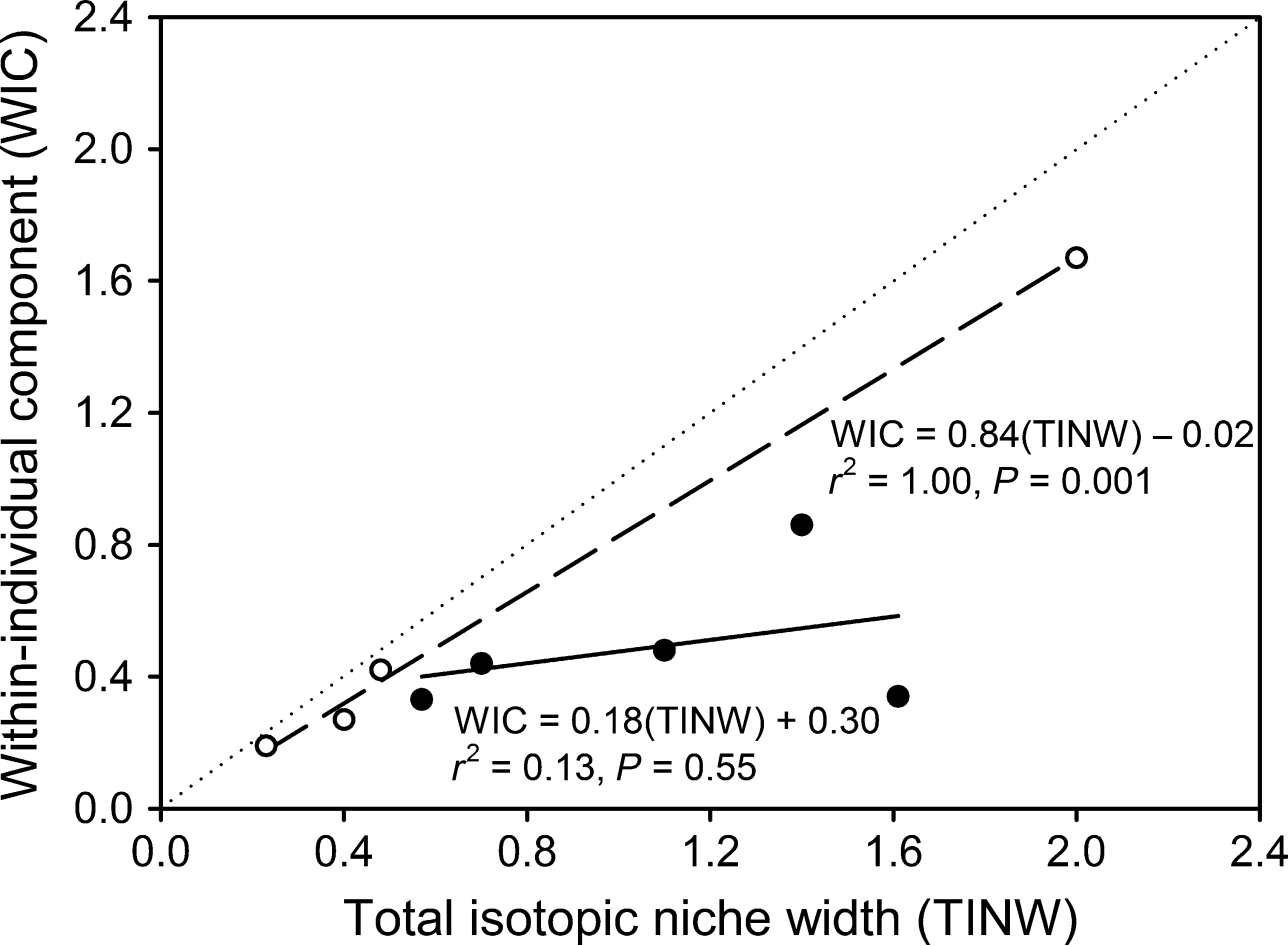
Linear regression between total isotopic niche width (TINW) and within‐individual component (WIC) for ringed seals (closed circles) and beluga whales (open circles). The slope for beluga whales (long‐dashed line) is significantly higher than that of ringed seals (solid line). The dotted line represents a 1:1 relationship.

A significant negative linear relationship between TINW and latitude occurred when both species were included in analyses (Fig. 3C; slope = −0.09, *r* ^2^ = 0.64, *F*_1,7_ = 12.24, *P* = 0.01), but not when species were run separately (*F*_1,5_ = 7.39, *P* = 0.07 for ringed seals, and *F*_1,3_ = 5.73, *P* = 0.14 for beluga). In addition, the WIC declined at a higher rate than BIC with increasing latitude, however was only marginally significant (Fig. 3A,B; WIC: slope = −0.05, *r* ^2^ = 0.41, *F*_1,7_ = 4.92, *P* = 0.06; BIC: slope = −0.03, *r* ^2^ = 0.25, *F*_1,7_ = 2.38, *P* = 0.17) and was largely influenced by the slope of the beluga data. When analyzed by species separately, WIC for beluga whales declined at a higher rate compared to ringed seals relative to latitude with slopes of −0.11 and −0.02, respectively, but neither was significant (beluga: *F*_1,3_ = 5.79, *P* = 0.14, and *F*_1,4_ = 2.33, *P* = 0.22 for ringed seals). For ringed seals, BIC declined at a higher rate than WIC relative to latitude (−0.04 and −0.02), but was not significant (*F*_1,4_ = 1.32, *P* = 0.33). The degree of IS (*i.e.,* WIC/TINW ratio) did not significantly change with increasing latitude (Fig. 3D; slope = 0.008, *r* ^2^ = 0.04, *F*_1,8_ = 0.28, *P* = 0.61). No significant relationships between WIC (*r* ^2^ = 0.04, *P* = 0.59), BIC (*r* ^2^ = 0.06, *P* = 0.53), TINW (*r* ^2^ = 0.10, *P* = 0.42), and WIC/TINW (*r* ^2^ < 0.01, *P* = 0.96) and longitude occurred when both species were combined. A significant relationship between WIC and TINW occurred for beluga (slope = 0.84, *r* ^2^ = 1.00, *F*_1,3_ = 774.6, *P* < 0.001; Fig. 4) and had a marginally significant higher slope than ringed seals (t_5_ = 2.55, *P* = 0.051). No significant relationship between WIC and TINW occurred for ringed seals (slope = 0.18, *r* ^2^ = 0.13, *F*_1,4_ = 0.45, *P* = 0.55; Fig. 4) or between the degree of IS and density among locations (slope = 0.04, *r* ^2^ = 0.06, *F*_1,3_ = 0.13, *P* = 0.75).

## Discussion

The TINW for ringed seal and beluga whale populations decreased with increasing latitude likely due to higher ecological opportunity in the low Arctic than the high Arctic. For both predator species, the increase in their TINW was mainly driven by *δ* ^15^N than *δ* ^13^C. In contrast to our hypothesis, the WIC of beluga increased in a near 1:1 relationship with TINW as all individuals within each population increased their niche breadth, suggesting that beluga whales, as a species, are dietary generalists. The slope between WIC and TINW for ringed seals was significantly lower than beluga, not significantly different from 0, and similar to relationships observed in “individual specialist” sea otters (*Enhydra lutris;* slope = 0.23; Newsome et al. 2015), implying a high degree of dietary individuality in populations of ringed seals which have a larger TINW possibly driven by ecological opportunity and being omnivorous. Despite relatively higher TINWs and more ecological opportunity at lower latitudes, the degree of IS (WIC/TINW) did not change with latitude for either species, contradictory to our hypothesis and the niche variation hypothesis. However, a high degree of IS occurred in ringed seals from Pangnirtung and Chesterfield Inlet, two of the low latitudinal sites. Other ecological factors, such as the intensity of interspecific and intraspecific competition and level of predation, may have driven the higher degree of IS for ringed seals at Pangnirtung and Chesterfield Inlet, which is explored in more detail below.

### Ecological opportunity

Spatial heterogeneity in a consumer's TINW respective to resource abundance and diversity has been observed in a variety of species ranging from invertebrates (Svanbäck et al. 2011) to vertebrates (Layman et al. 2007; Darimont et al. 2009). The trophic dynamics of Arctic regions at southerly latitudes have been changing due to the recent northward range expansion of subarctic fish and plankton species (Wassmann et al. 2011) where seabirds have shifted their diet from Arctic cod to Capelin and Sand lance at lower latitudes (Provencher et al. 2012). In our study, ringed seals and beluga had larger TINWs at lower latitudes as a likely response to increased ecological opportunity. This result is further supported by longitude having no site‐specific significant effect on any of the niche metrics for both species. Spatial differences in beluga whale diet have been reported with individuals mainly consuming highly abundant Arctic cod in the high Arctic locations of the Beaufort Sea (Loseto et al. 2009) and Resolute (Matley et al. 2015). At lower latitudes, beluga whales now consume other pelagic fish species including Capelin and Sand lance near Pangnirtung (*i.e.,* Cumberland Sound; Marcoux et al. 2012) and Hudson Bay (Kelley et al. 2010). Similarly, ringed seals have been reported to mainly consume Arctic cod in the high Arctic with higher dietary proportions of Capelin, Sand lance, and invertebrates at lower latitudes (Yurkowski et al. in press). The combination of a high WIC/TINW ratio and a low TINW for ringed seals and beluga whales inhabiting the high Arctic suggests dietary specialization at the population level where each species only consumes one prey type or functional group in this case being pelagic forage fish, mainly Arctic cod.

The ecological opportunity concept is related to interspecific competition and its effects on niche width and individual specialization in consumer populations, in that an increase in ecological opportunity or a decrease in interspecific competition promotes larger population niche widths and IS among individuals (Bolnick et al. 2010; Araújo et al. 2011). With WIC having a steeper slope than BIC relative to latitude and WIC significantly increasing with TINW in beluga whales, this suggests a parallel ecological release where both the individual and population niche widths increase in similar proportions in response to novel prey types (Bolnick et al. 2010). A similar result occurred in female Antarctic fur seals (*Arctocephalus gazelle*) where they increased population TINW by enlarging their individual niche breadth during the interbreeding period when females typically gain condition by foraging intensively after weaning (Kernaléguen et al. 2015). Moreover, a similar relationship between WIC and TINW (slope = 0.54) occurred in sea otter populations from the mixed substrates where all individuals utilized multiple prey types or functional groups (Newsome et al. 2015).

Consistent with the niche variation and between‐individual niche variation hypotheses, the BIC had a steeper slope than WIC relative to latitude and contributed more to higher TINW values than WIC in ringed seals. A comparable result where a higher TINW corresponded to higher interindividual variation and a high degree of IS occurred in several other vertebrate species, including fruit bats (*Rousettus aegyptiacus*; Herrera et al. 2008), green turtles (*Chelonia mydas*; Vander Zanden et al. 2010), brown trout (*Salmo trutta*; Evangelista et al. 2014), gray snappers (*Lutjanus griseus*; Layman et al. 2007), gray wolves (*Canis lupus*; Darimont et al. 2009), sea otters (Newsome et al. 2015), and subantarctic fur seals (*Arctocephalus tropicalis*; Kernaléguen et al. 2015). With the preponderance of subarctic species inhabiting the low Arctic, ringed seals have the opportunity to forage upon more prey types and functional groups by increasing their niche size and degree of trophic omnivory (Yurkowski et al. in press), thereby increasing interindividual variation. Despite a higher BIC at relatively lower latitudes, the degree of IS in ringed seals did not significantly change with latitude, but was observed to be highest in Chesterfield Inlet and Pangnirtung, two geographic areas where non‐native Sand lance and Capelin have become common (Marcoux et al. 2012; Provencher et al. 2012). Consequently, some of the site‐specific variations in IS may not be solely predicted by ecological opportunity, as the level of intraspecific and interspecific competition for resources and predation pressure likely has influence at both locations (Svanbäck and Bolnick 2005, 2007; Bolnick et al. 2010). The effect of interspecific competition could not be interpreted due to a lack of any accurate data on the abundance or density of subarctic mammals, such as harbor seals (*Phoca vitulina*) and harp seals (*Pagophilus groenlandicus*) at each geographic location, but both species have been reported to be increasing in abundance in Hudson Bay and Cumberland Sound (Diemer et al. 2011; Bajzak et al. 2012).

### Intraspecific competition

Strong intraspecific competition from high densities of a population can lead to a broader population niche width and a higher degrees of IS among individuals (Svanbäck and Bolnick 2005; Evangelista et al. 2014), but can also reduce interindividual variation and degree of IS as all individuals may converge onto an alternative prey resource due to changes in the preferred primary prey resource (Araújo et al. 2011). Densities have not been estimated for beluga whales near each sampling location, so we used total abundance estimates to provide a tentative assessment on the influence of intraspecific competition for resources on TINW and degree of IS. Intraspecific competition may have partially contributed to a higher TINW in beluga whales from Arviat, as abundance was highest in Western Hudson Bay (57,300; Richard 2005) compared to Eastern Beaufort and Chukchi Seas (42,958; Frost et al. 1993; Allen and Angliss 2011), areas encompassing Barrow Strait near Resolute (21,200; Innes et al. 2002) and Cumberland Sound (1,547; COSEWIC 2004). In contrast to our hypothesis, the degree of individual specialization (WIC/TINW) for beluga was low (≥0.68) among all locations regardless of varying beluga abundances, suggesting that all beluga individuals expand their niche and diverge on a similar prey functional group, most likely pelagic forage fish (Loseto et al. 2009).

Density estimates for ringed seals vary interannually, but were much higher in the Amundsen Gulf area near Ulukhaktok ranging from 2 to 3.5 seals/km^2^ in 1984 (Kingsley 1986) and Baffin Bay in 1978–1979 (2.8 seals/km^2^; Kingsley 1998) near Cumberland Sound compared to Resolute (ranging from 0.21 to 1.16 seals/km^2^ in 1980–1982, average = 0.57 seals/km^2^; Kingsley et al. 1985) and Western Hudson Bay (ranging from 0.20 to 1.22 seals/km^2^ in 1995–2013, average = 0.65 seals/km^2^; Young et al. in press). Abundance or density estimates for ringed seals have not been conducted near the Labrador region encompassing Saglek Bay. No discernable relationship between ringed seal density and TINW or IS was apparent, in contrast to our hypothesis and previous studies where higher densities (*i.e.,* intraspecific competition) of consumer populations lead to a higher degree of TINW and IS (Svanbäck and Bolnick 2007; Evangelista et al. 2014; Newsome et al. 2015). Along with increased ecological opportunity, higher ringed seal density in Baffin Bay may have contributed to a broader population niche width and a higher level of IS in ringed seals near Pangnirtung. Consistent with optimal diet theory (Schoener 1971), all individuals have a preferred prey resource, in this case likely being energy‐rich Arctic cod (24.2 kJ/g/dw; Weslawski et al. 1994). But differences in rank‐preference variation for alternative resources among individuals, such as invertebrates (12.3–21.1 kJ/g/dw; Weslawski et al. 1994) and Capelin (21.2 kJ/g/dw; Hedeholm et al. 2011), can lead to increased population niche widths and higher levels of IS among individuals, which was also observed in subantarctic fur seals (Kernaléguen et al. 2015). Alternatively, the highest level of IS for ringed seals occurred in Western Hudson Bay – an area of relatively lower ringed seal density and high ecological opportunity, suggesting that individuals within the population may already have distinct preferred prey resources (Araújo et al. 2011). However, the high degree of IS for Western Hudson Bay ringed seals may also be influenced by other ecological factors, such as decreased predation pressure.

The effect of decreased predation pressure from polar bears (*Ursus maritimus*), the main predator of ringed seals (Stirling and Derocher 2012), could be associated with the higher degree of IS of ringed seals from Baffin Bay and Western Hudson Bay, as both polar bear populations have declined (Regehr et al. 2007; Laidre et al. 2015; Lunn et al. 2015). Increased predation pressure has been shown to decrease IS (Eklöv and Svanbäck 2006); thus, decreased predation pressure potentially allows ringed seal individuals to be more risk averse, thereby increasing their level of IS among individuals and, in turn, their population niche width. In addition, the Davis Strait polar bear population that encompasses Saglek Bay is stable (Laidre et al. 2015) and would likely have relatively higher predation pressure which may influence the low degree of IS for Saglek Bay ringed seals.

### Summary

The TINW for ringed seal and beluga decreased with increasing latitude most likely due to an increased ecological opportunity at lower latitudes. However, the relationship between individual niche metrics (WIC and BIC) and TINW, as well as latitude, differed between both species where in ringed seals, BIC contributed more than WIC to higher TINW values implying individuality in ringed seals. In beluga, WIC increased in a near 1:1 ratio with TINW suggesting dietary generalization. The effect of intraspecific competition on TINW and the degree of IS were mixed, but no relationship between TINW or the degree of IS and consumer density was apparent for both species. In concordance with the results from this study, Svanbäck et al. (2011) reported that resource abundance, not consumer density (*i.e.,* intraspecific competition), was the main component driving a higher TINW and degree of IS. The influence of ecological opportunity affecting niche metrics and IS in animals is likely underrepresented in the ecological literature as most studies have primarily investigated the effects of intraspecific and interspecific competition on niche variability and the degree of IS. In conclusion, latitudinal differences in niche metrics between beluga whales and ringed seals relative to ecological opportunity and intraspecific competition suggested the species‐specific variation in the ability for dietary plasticity to changing resource and environmental conditions in the Arctic.

## Data Accessibility

Data supporting our results is archived in the Dryad public archive (datadryad.org). Dryad Digital Repository. doi:10.5061/dryad.4j8j2

## Conflict of Interest

None declared.

## Supporting information


**Appendix S1.** Parameter estimates from linear mixed‐models for ringed seal δ^13^C and δ^15^N values at each location relative to age class, sex, standard length, tissue and year collected with seal ID as a random effect.

**Appendix S2.** Parameter estimates from linear mixed‐models for beluga whale δ^13^C and δ^15^N values at each location relative to age class, sex, standard length, tissue and year collected with ID as a random effect.
