# Supplementary material for: Latitudinal variation in ecological opportunity and intraspecific competition indicates differences in niche variability and diet specialization of Arctic marine predators
Source: Ecol Evol. 2016 Feb 14;6(6):1666–78. doi: 10.1002/ece3.1980 (PMC4752956; doi:10.1002/ece3.1980)
Supplement: Supplementary file 1 — Appendix S1. Parameter estimates from linear mixed‐models for ringed seal δ13C and δ15N values at each location relative to age class, sex, standard length, tissue and year collected with seal ID as a random effect. [file ECE3-6-1666-s001.docx]

**Appendix S1.** Parameter estimates from linear mixed-models for ringed seal δ^13^C and δ^15^N values at each location relative to age class, sex, standard length, tissue and year collected with seal ID as a random effect. Significant p-values are highlighted in bold. SE: Standard error

|  |  | δ^13^C (‰) | | | |  | δ^15^N (‰) | | | |
| --- | --- | --- | --- | --- | --- | --- | --- | --- | --- | --- |
|  | Predictor variable | Slope ± SE | t-statistic | df | p-value |  | Slope ± SE | t-statistic | df | p-value |
| **Chesterfield Inlet** | |  |  |  |  |  |  |  |  |  |
|  | Age class | -0.13 ± 0.27 | -0.47 | 29 | 0.64 |  | 0.60 ± 0.48 | 1.24 | 29 | 0.22 |
|  | Sex | -0.15 ± 0.21 | -0.70 | 29 | 0.49 |  | -0.58 ± 0.37 | -1.58 | 29 | 0.12 |
|  | Standard length | 0.02 ± 0.008 | 2.85 | 29 | **<0.01** |  | 0.03 ± 0.01 | 2.26 | 29 | **0.03** |
|  | Year | 0.31 ± 0.21 | 1.42 | 29 | 0.17 |  | 0.28 ± 0.38 | 0.74 | 29 | 0.46 |
|  | Tissue | -0.67 ± 0.08 | -8.69 | 33 | **<0.001** |  | 0.51 ± 0.12 | 4.23 | 33 | **<0.001** |
| **Pangnirtung** | |  |  |  |  |  |  |  |  |  |
|  | Age class | 0.28 ± 0.13 | 2.16 | 72 | **0.03** |  | -0.66 ± 0.19 | -3.49 | 72 | **<0.001** |
|  | Sex | -0.27 ± 0.15 | -1.29 | 72 | 0.20 |  | 0.11 ± 0.17 | 0.66 | 72 | 0.51 |
|  | Standard length | 0.01 ± 0.005 | 2.40 | 72 | **0.02** |  | 0.005 ± 0.006 | 0.81 | 72 | 0.42 |
|  | Year | -0.008 ± 0.01 | -0.61 | 72 | 0.55 |  | -0.009 ± 0.02 | -0.47 | 72 | 0.64 |
|  | Tissue | -0.76 ± 0.05 | -15.36 | 76 | **<0.001** |  | 0.42 ± 0.10 | 4.11 | 76 | **<0.001** |
| **Resolute** | |  |  |  |  |  |  |  |  |  |
|  | Age class | -0.27 ± 0.15 | -1.78 | 41 | 0.08 |  | -0.59 ± 0.21 | -2.76 | 41 | **<0.01** |
|  | Sex | -0.16 ± 0.13 | -1.19 | 41 | 0.24 |  | 0.20 ± 0.19 | 1.06 | 41 | 0.30 |
|  | Standard length | 0.004 ± 0.003 | 1.53 | 41 | 0.13 |  | -0.004 ± 0.004 | -0.94 | 41 | 0.35 |
|  | Year | 0.001 ± 0.02 | 0.05 | 41 | 0.96 |  | -0.06 ± 0.03 | -1.80 | 41 | 0.08 |
|  | Tissue | -0.82 ± 0.07 | -12.10 | 45 | **<0.001** |  | 0.04 ± 0.12 | 0.36 | 45 | 0.72 |
| **Saglek Bay** | |  |  |  |  |  |  |  |  |  |
|  | Age class | 0.17 ± 0.26 | 0.65 | 64 | 0.52 |  | 0.85± 0.37 | 2.27 | 64 | **0.03** |
|  | Sex | 0.02 ± 0.14 | 0.16 | 64 | 0.87 |  | -0.07 ± 0.20 | -0.36 | 64 | 0.72 |
|  | Standard length | 0.002 ± 0.007 | 0.26 | 64 | 0.80 |  | 0.04 ± 0.01 | 3.72 | 64 | **<0.001** |
|  | Year | -0.10 ± 0.07 | -1.50 | 64 | 0.14 |  | 0.21 ± 0.10 | 2.18 | 64 | **0.03** |
|  | Tissue | -0.61 ± 0.11 | -5.47 | 68 | **<0.001** |  | 0.96 ± 0.11 | 8.89 | 68 | **<0.001** |
| **Ulukhaktok** | |  |  |  |  |  |  |  |  |  |
|  | Age class | 0.12 ± 0.14 | 0.91 | 149 | 0.36 |  | -0.47 ± 0.19 | -2.50 | 149 | **0.01** |
|  | Sex | 0.02 ± 0.07 | 0.22 | 149 | 0.83 |  | 0.19 ± 0.10 | 1.99 | 149 | **<0.05** |
|  | Standard length | 0.004 ± 0.004 | 1.09 | 149 | 0.28 |  | 0.02 ± 0.005 | 4.08 | 149 | **<0.001** |
|  | Year | -0.01 ± 0.007 | -2.17 | 149 | **0.03** |  | -0.001 ± 0.009 | -0.14 | 149 | 0.89 |
|  | Tissue | -0.19± 0.05 | -4.20 | 153 | **<0.001** |  | -0.08± 0.05 | -1.62 | 153 | 0.11 |
