# Supplementary material for: Latitudinal variation in ecological opportunity and intraspecific competition indicates differences in niche variability and diet specialization of Arctic marine predators
Source: Ecol Evol. 2016 Feb 14;6(6):1666–78. doi: 10.1002/ece3.1980 (PMC4752956; doi:10.1002/ece3.1980)
Supplement: Supplementary file 2 — Appendix S2. Parameter estimates from linear mixed‐models for beluga whale δ13C and δ15N values at each location relative to age class, sex, standard length, tissue and year collected with ID as a random effect. [file ECE3-6-1666-s002.docx]

**Appendix S2.** Parameter estimates from linear mixed-models for beluga whale δ^13^C and δ^15^N values at each location relative to age class, sex, standard length, tissue and year collected with ID as a random effect. Significant p-values are highlighted in bold. HI: Hendrickson Island, SE: Standard error

|  |  | δ^13^C (‰) | | | |  | δ^15^N (‰) | | | |
| --- | --- | --- | --- | --- | --- | --- | --- | --- | --- | --- |
|  | Predictor variable | Slope ± SE | t-statistic | df | p-value |  | Slope ± SE | t-statistic | df | p-value |
| **Resolute** | |  |  |  |  |  |  |  |  |  |
|  | Sex | 0.11 ± 0.24 | 0.47 | 7 | 0.65 |  | 0.07 ± 0.28 | 0.25 | 7 | 0.81 |
|  | Standard length | 0.002 ± 0.002 | 0.90 | 7 | 0.40 |  | 0.003 ± 0.003 | 1.28 | 7 | 0.25 |
|  | Year | 0.02 ± 0.02 | 0.66 | 7 | 0.53 |  | 0.006 ± 0.03 | 0.21 | 7 | 0.84 |
|  | Tissue | 0.32 ± 0.08 | 3.89 | 10 | **<0.01** |  | -0.15 ± 0.17 | -0.88 | 10 | 0.40 |
| **HI/Paulatuk** | |  |  |  |  |  |  |  |  |  |
|  | Standard length | 0.001± 0.003 | 0.45 | 29 | 0.65 |  | 0.002 ± 0.003 | 0.94 | 29 | 0.36 |
|  | Year | 0.01 ± 0.14 | 0.09 | 29 | 0.93 |  | 0.09± 0.12 | 0.78 | 29 | 0.45 |
|  | Tissue | -1.47 ± 0.09 | -16.14 | 31 | **<0.001** |  | -1.58 ± 0.09 | -16.63 | 31 | **<0.001** |
| **Pangnirtung** | |  |  |  |  |  |  |  |  |  |
|  | Age class | -0.20 ± 0.12 | -1.75 | 26 | 0.09 |  | -0.07 ± 0.22 | -0.32 | 26 | 0.75 |
|  | Sex | -0.04 ± 0.10 | -0.37 | 26 | 0.72 |  | 0.22 ± 0.19 | 1.17 | 26 | 0.25 |
|  | Standard length | 0.001 ± 0.001 | 1.01 | 26 | 0.32 |  | 0.007 ± 0.002 | 3.22 | 26 | **<0.01** |
|  | Year | -0.02 ± 0.01 | -2.67 | 26 | 0.01 |  | -0.10 ± 0.01 | -7.40 | 26 | **<0.001** |
|  | Tissue | -0.94 ± 0.04 | -20.98 | 30 | **<0.001** |  | -2.37 ± 0.16 | -14.87 | 30 | **<0.001** |
| **Arviat** | |  |  |  |  |  |  |  |  |  |
|  | Age class | 0.48 ± 0.24 | 2.00 | 39 | 0.05 |  | -0.35 ± 0.36 | -0.97 | 39 | 0.34 |
|  | Sex | 0.16 ± 0.20 | 0.80 | 39 | 0.43 |  | 0.69 ± 0.30 | 2.30 | 39 | **0.03** |
|  | Standard length | 0.002 ± 0.002 | 1.25 | 39 | 0.22 |  | -0.0003 ± 0.003 | -0.11 | 39 | 0.91 |
|  | Year | 0.04 ± 0.05 | 0.82 | 39 | 0.42 |  | 0.04 ± 0.08 | 0.52 | 39 | 0.60 |
|  | Tissue | -1.39 ± 0.15 | -9.31 | 43 | **<0.001** |  | -2.03 ± 0.29 | -7.09 | 43 | **<0.001** |
